# Adaptive Changes in the Dynamics of Visual Attention With Extended Practice

**DOI:** 10.3389/fpsyg.2020.565288

**Published:** 2020-10-06

**Authors:** Matthew S. Junker, Bo Youn Park, Jacqueline C. Shin, Yang Seok Cho

**Affiliations:** ^1^School of Psychological and Behavioral Sciences, Southern Illinois University, Carbondale, IL, United States; ^2^Department of Psychology, Korea University, Seoul, South Korea; ^3^Department of Psychology, Indiana State University, Terre Haute, IN, United States

**Keywords:** temporal attention, attentional dynamics, explicit learning, attentional control, procedural learning

## Abstract

Previous research indicates that visual attention can adapt to temporal stimulus patterns utilizing the rapid serial visual presentation (RSVP) task. However, how the temporal dynamics of an attentional pulse adapt to temporal patterns has not been explored. We addressed this question by conducting an attentional component analysis on RSVP performance and explored whether changes in attentional dynamics were accompanied by explicit learning about predictable target timing. We utilized an RSVP task in which a target letter appeared either in two possible RSVP positions in fixed-timing conditions or in random positions over 1, 2, or 3 days of training. In a transfer phase, the target appeared in previously presented or new positions. Over 3 days of practice the target identification rate, efficacy, and precision of a putative attentional pulse increased. These changes reflected general learning in the RSVP task resulting in attentional dynamics more efficiently focused on the target. Although group performance effects did not support learning of fixed target positions, target identification rates and the measure of the efficacy of an attentional pulse at these positions were positively associated with explicit learning. The current study is the first to provide a detailed description of practice related adaptation of attentional dynamics and suggests that timing specific changes might be mediated by explicit temporal learning.

## Introduction

The ability for human beings to adapt to environmental change is essential for survival. Because many elements in dynamic environments have predictable or rhythmic patterns, such as in movement coordination and daily conversation, the ability to learn the temporal pattern of events would be adaptive (e.g., Barnes and Jones, 2000; Shin and Ivry, 2002; Miller et al., 2013). Essential to temporal adaptation is the ability to adjust attentional dynamics in a way that corresponds to the temporal pattern of environmental events. By attentional dynamics, we refer to the distribution of attentional resources over time, as opposed to the distribution of attentional resources over space. This conceptualization complements approaches that conceptualize the spatial adaptation of visual attention using “spotlight” metaphors (Posner et al., 1980) by emphasizing the time dimension. We emphasize a “pulse” metaphor of attention (Reeves and Sperling, 1986; Barnes and Jones, 2000). The current work takes a novel approach focusing on how the temporal dynamics of attention change with training in the presence of consistent temporal information.

Attentional adjustments to temporally patterned stimuli would be adaptive since perceptual and short-term memory capacity is limited, making it necessary for humans to prioritize and select from an overwhelming amount of environmental information. Much previous research highlights our ability to select crucial goal-related information to enhance the efficiency of perceptual and memory processes. Selective attention enhances the perceptual processing of visual stimuli held briefly in sensory memory and facilitates the transfer of that perceptual information to short-term memory (Reeves and Sperling, 1986; Dell’Acqua et al., 2010; Murray et al., 2011; Hilkenmeier et al., 2012). Evidence for perceptual enhancement with attentional selection in the spatial domain is found in cue validity effects (Bashinski and Bacharach, 1980; Posner, 1980; Yeshurun and Carrasco, 1999; Carrasco et al., 2000; Lu and Dosher, 2000), attentional capture (Yantis and Jonides, 1984; Theeuwes, 1994), and enhanced processing of stimuli of statistical regularities that drive attentional expectations (Mayr, 1996; Chun, and Jiang, 1999). Similar mechanisms operate in the temporal domain with evidence for cue validity and attentional expectancies that are driven by consistent fore-periods (Niemi and Näätänen, 1981; Coull and Nobre, 1998; Correa et al., 2005, 2006) and periodicity in stimulus presentation (Jones et al., 1982, 2002; Large and Jones, 1999; Miller et al., 2013).

Recent work on temporal attention focuses on the skill that develops through repeating temporal patterns using the rapid serial visual presentation (RSVP) paradigm (Tang et al., 2014; Shin et al., 2015). In an RSVP task, visual stimuli are presented at the rate of 10–20 Hz. The body of work using the RSVP task reveals limitations in the rate at which multiple visual targets can be encoded as distinctive stimuli into working memory, represented by the *attentional blink* (*AB*) phenomenon (Broadbent and Broadbent, 1987; Raymond et al., 1992). The AB, defined as a reduction in identification of a target (*T2*) that is preceded by another target (*T1*) by about 200–500 ms, reveals that capacity limited attentional processes necessary to encode information into visual working memory have a temporal time course that takes up to 500–600 ms to complete. Furthermore, if no non-target stimuli occur between T1 and T2, participants will often report T2 (Raymond et al., 1992; Hommel and Akyürek, 2005) as well as any additional targets that follow without an intervening non-target distractor (Olivers et al., 2007). However, this attenuation of the AB when intervening stimuli are not present is accompanied by the loss of temporal information concerning the order of targets, suggesting that the process of binding temporal information to distinctive stimuli causes the AB (Wyble et al., 2009). The ability of distractor stimuli to influence the AB highlights the malleability of temporal attentional dynamics. Several recent studies revealed that AB could also be reduced with practice (Choi et al., 2012; Tang et al., 2014; Shin et al., 2015; Willems et al., 2015), underlining the malleability of AB with learning and challenging the initial construal of AB as reflecting a rigid bottleneck inherent in the attentional system (Chun and Potter, 1995; Jolicœur and Dell’Acqua, 1998; Dell’Acqua et al., 2012; Dux et al., 2014). In particular, a fixed temporal position of T2 relative to T1 leads to enhanced T2 identification, showing that attentional dynamics can adapt to temporal patterns (Martens and Johnson, 2005; Tang et al., 2014; Shin et al., 2015; Willems et al., 2015). Similarly, the reduction of AB when a periodic rhythm is presented concurrently with an RSVP stream (Ronconi et al., 2016a,b) suggests that periodicity is one such temporal pattern that attentional dynamics can adapt to.

The main goal of this study was to directly characterize the time course with which an attentional episode may rise and fall in intensity and observe how this time course changes as people adapt to temporal patterns. To do this, it was necessary to modify the basic RSVP paradigm and methods of measuring RSVP performance. The focus on measuring AB magnitude in the studies exploring learning effects makes it difficult to observe the time scale of attentional oscillations (Garner et al., 2014; Willems et al., 2015). Thus, it would be important to use RSVP tasks that do not demand the report of more than one target. Therefore, we deviated from standard practice and utilized single-target RSVP tasks.

The direct methods we used to measure the time course of attentional dynamics was inspired by the work of Sperling and colleagues (Reeves and Sperling, 1986; Weichselgartner and Sperling, 1987; Shih and Sperling, 2002) and Vul et al., (2008) in their methods of characterizing an “attentional pulse.” By manipulating various spatial and temporal properties of visuospatial arrays in RSVP tasks, Sperling and colleagues tested an *attentional gating model* of temporal attentional dynamics in which visual information in sensory storage is stochastically selected and transferred into short-term memory. The dynamic profile of attention as it was directed toward the target was described as a rising and falling of an attentional “pulse.” Adapting this conceptualization of temporal attention to the AB task, Vul et al., (2008) computed parameters of a putative attentional pulse that occurred for the processing of T2 in the AB task. Their measure of *efficacy* characterized how much attention was allocated to a brief time window surrounding a target stimulus. Their measure of *Latency* characterized when, relative to a target, more attention was allocated (before or after the target). Finally, their measure of *Precision* characterized how focused attention was on the target. These computations capitalized on the temporal distribution of intrusion errors for T2 report centering on the immediate serial positions surrounding T2. In the current study, we had participants perform a single target RSVP task for varying numbers of practice sessions and applied the same type of analyses as Vul et al., (2008). Details for calculating these parameters are outlined in the Section “Materials and Methods.”

In addition to adapting Vul et al., (2008) parameters to a single target RSVP paradigm task, we varied whether the target position was predictable or unpredictable. Our goal was to characterize how the parameters of an attentional pulse described above changed as a function of practice, which were varied at one, two, or three sessions of RSVP practice completed over successive days. Enhancements in RSVP performance would be indicated by observing changes in attentional dynamics that occurred during a practice phase and by detecting differences in attentional dynamics during a transfer phase in which both practiced and new target positions were presented.

We expected that participants would be able to adaptively change their attentional dynamics to improve task performance as measured by the target identification rate. Presumably, target identification rate would increase as a function of attentional resources that peaked closely around the target itself. Thus, it was predicted that the efficacy would increase, and the variability would decrease with practice. Still assuming that maximization of target identification performance would involve the peaking of an attentional pulse closely around the target, we also predicted the latency would approach the target position with practice.

We adopted the working hypothesis that this attunement of attention involved a procedural learning process. However, the implicit or explicit nature of attentional attunement is by no means obvious given the wide range of task demands in RSVP studies demonstrating attentional attunement. While AB could be reduced under implicit learning conditions where explicit knowledge of the predictable time constraints was minimized, this was only evidenced with a very large number of trials (around 1500) (Shin et al., 2015). In contrast, AB reduction was reliably observed in multiple studies with relatively small or medium amounts of practice under explicit learning conditions (Martens and Johnson, 2005; Tang et al., 2014). It is important to note that virtually none of the research with fixed target serial positions showing effects of practice has systematically distinguished the effects of explicit and implicit learning (e.g., Tang et al., 2014; Visser et al., 2014, 2015). One of our goals was to examine whether explicit knowledge of temporal patterns emerged with practice and whether this knowledge was associated with changes in the dynamics of an attentional pulse. In the current study, explicit learning is said to have occurred if participants are conscious of the temporal positions of the fixed target serial positions at the end of the experiment. This definition of explicit learning differs from previous work which manipulates participants’ explicit knowledge of target timing with informative cues provided prior to the RSVP stream (Martens and Johnson, 2005). Specifically, we measured the degree of explicit learning for individual participants using a questionnaire administered at the end of the experiment and examined the relationship between the degree of explicit learning and the changes in target identification performance and parameters of attentional dynamics. We expected participants who explicitly learned the target timing to adjust attentional dynamics faster than those who demonstrated less explicit learning of target timing.

## Materials and Methods

### Participants

The participants were 135 undergraduate and graduate students at Korea University (*F* = 67, *M* = 68) ranging in age from 18 to 33 years (*M* = 22.9, *SD* = 2.79). Informed consent, approved by the Institutional Review Board of Korea University (KU-IRB-16-138-A-1), was given at the beginning of the experiment. All participants reported normal or corrected-to-normal vision.

### Stimuli

Stimuli were presented on a 15.9 in CRT monitor with an 85 Hz graphics card using the *E-Prime 2.0* program. The room was dimly lit with a lamp angled horizontally toward the wall directly centered behind the monitor. A chin rest was fixed at a distance of 60 cm away from, and directly centered in front of, the monitor to ensure that participants maintained a constant viewing distance.

Stimuli were uppercase letters of the English alphabet, excluding *I*, *O*, *U*, and *V*. Sixteen of the remaining 22 letters were randomly selected each trial and presented sequentially in the center of the screen. All stimuli were in 13 point Arial font, resulting in a visual angle of approximately 0.51° by 0.51°. Distractor stimuli were presented in white, and target stimuli were presented in blue, both presented on a gray background.

### Procedure

After signing the informed consent form, participants were seated at the computer, where they reported their age and sex. Participants then read a set of instructions. The instructor remained in the room while the participants completed 9 practice trials.

**FIGURE 1:**
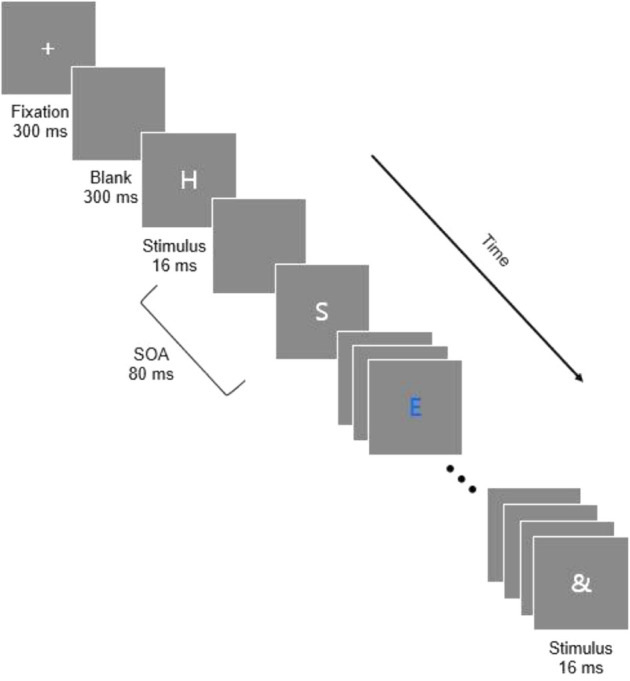
The sequence of stimulus events within a stream for the RSVP task. Each RSVP stream contained 16 letters. The identities of the target and distractors varied from trial to trial. In these examples, the target is the letter *E* in blue.

Participants began each trial by pressing the space bar. After they pressed the space bar, a 300 ms blank display was followed by a fixation cross ‘+’ lasting 300 ms. Another blank display of 300 ms preceded the onset of the first letter in the RSVP stream. Figure 1 depicts the sequence of events which occurred in a trial.

Sixteen letters were presented on each trial, and each block contained 54 trials. Forty-eight of the 54 trials in a block included one target and 15 distractors, and 6 of the trials in each block were catch trials consisting of only 16 distractors without a target. The stimuli were programmed to be presented sequentially in the center of the screen with a *stimulus-onset asynchrony* (*SOA*) of 80 ms and a display time of 16 ms, resulting in a blank inter-stimulus interval (ISI) of 64 ms. However, due to the limitations of the 85 Hz graphics card, the actual timings of stimulus presentation were 82.5 ms SOA with a stimulus duration of 23.53 ms and an ISI of 58.8 ms. We believe, however, that this discrepancy in stimulus timing does not critically change the interpretation of the results. At the end of each stream of stimuli, an and mask was presented for 16 ms. At the end of the RSVP stream, a screen appeared that asked participants to report the blue target letter. Participants were instructed to use their best guess if they thought that there was a blue letter but were unsure what it was. Participants responded using the *0* key to indicate that there was no blue letter if they thought a trial was a catch trial. The *Enter* key was used to submit a response and then participants pressed the space bar to begin the next trial. Blocks of trials were separated by a forced 30 s break.

For each participant, the RSVP task was practiced in two phases–*training* and *transfer*. The possible RSVP positions of the targets on target-present trials during training varied among participant groups in different *Target Timing* conditions. In the *4–10* condition, the target was equally likely to appear at Positions 4 and 10. In the *7–13* condition, the target was equally likely to appear at Positions 7 and 13. In the *random* condition, the target was equally likely to appear at Position 3 to Position 14 on a given trial.

In addition, *Training Level* was manipulated between participants. Participants performed the RSVP task during one experimental session (*1-Day* condition), two experimental sessions over consecutive days (*2-Day* condition), or three experimental sessions over consecutive days (*3-Day* condition). The number of training blocks was 3, 9, and 15 for the 1-Day, 2-Day, and 3-Day conditions, respectively. The last day of training contained 3 training blocks, and all other days contained 6 training blocks each. Target Timing and Training Level were combined in a factorial design yielding nine between-subjects conditions, with 15 participants randomly assigned to each group.

After the training phase on the last day of training, all participants performed 3 blocks in a transfer phase. Regardless of Target Timing and Training Level, the target appeared with equal frequency at Positions 4, 7, 10, and 13 during each block of 54 trials. Both training and transfer blocks included the same number of target-present and catch trials.

After completing the final session, participants were given the Questionnaire of Explicit Learning.

#### Questionnaire of Explicit Learning

This questionnaire assessed explicit knowledge of the temporal positions of the targets with respect to both the training phase and the transfer phase. Participants first answered questions regarding the training phase; then they answered questions regarding the transfer phase. Finally, participants filled in a table with sixteen empty boxes, labeled from 1 to 16, with information about the relative frequency of target presentation at each serial position. Participants were allowed to indicate the percentages of trials or the absolute frequencies of target presentation. These numbers were normalized relative to the total indicated percentages or frequencies over the entire range of serial positions and used to measure the degree of explicit temporal knowledge gained through practice.

#### Measures of RSVP Performance, Attentional Dynamics, and Explicit Learning

Our main measure of RSVP performance was target identification rate, defined as the proportion of trials that the target letter was accurately reported at a given serial position. Attentional dynamics were measured by the parameters used in Vul et al., (2008)–*A values*, *C values*, and *V values*, which were defined using reports of letters as targets within a seven-stimulus window of the actual target letter (three targets immediately preceding the targets, the target, and three targets immediately following the target). *A* values were defined as the proportion of trials that a participant reported a letter which appeared within the seven-stimulus window and represent the *efficacy* with which attention is temporally focused around the target. *C* values were defined as the average distance (measured in number of serial positions) from the target that the reported letters occurred and quantifies the *latency* of attention relative to the target position. *V* values were defined as the variance of the serial positions of reported letters within the seven-stimulus window and signify the *precision* of a putative attentional pulse within that seven-stimulus window. See Vul et al., (2008) for detailed computational formulas.

Measures of explicit learning were computed from responses in the Questionnaire for Explicit Learning. Two sets of *Explicit Learning Score*s (*ELS*s) were computed for each participant–one relating to knowledge about target timing during the training phase and one relating to knowledge about target timing during the transfer phase. In each case, ELS was computed for all four positions in the RSVP task. Specifically, for Position *x*, the ELS was computed as the sum of the frequency responses for serial positions, *x* − 1, *x*, and *x* + 1 divided by the total frequency responses for all 16 serial positions. Therefore, for a given position, the baseline ELS would be 0.1875 (i.e., 3/16). Three serial positions were used rather than only one because the speed of the RSVP task would make it extremely difficult to identify target positions accurately.

## Results

The results focus on general learning in the RSVP task, timing specific learning, and the results regarding the relationship between explicit learning and attentional adaptation.

We excluded data from participants who showed high proportions of responses that indicated no target was presented during target-present trials by entering a *0* response (8 participants with more than 30% *0* responses to target-present trials). For each of the measures, we excluded participants whose mean was beyond two standard deviations from the Target Timing (4–10, 7–13, and random) and Training Level (1, 2, and 3 days of practice) group means. The reported results focus on effects whose level of significance were smaller than *p* < 0.05. Additionally, when necessary for repeated measures analyses, Greenhouse-Geisser corrections were implemented.

### General RSVP Learning: Monotonic Learning Curves During the Training Phase

**FIGURE 2:**
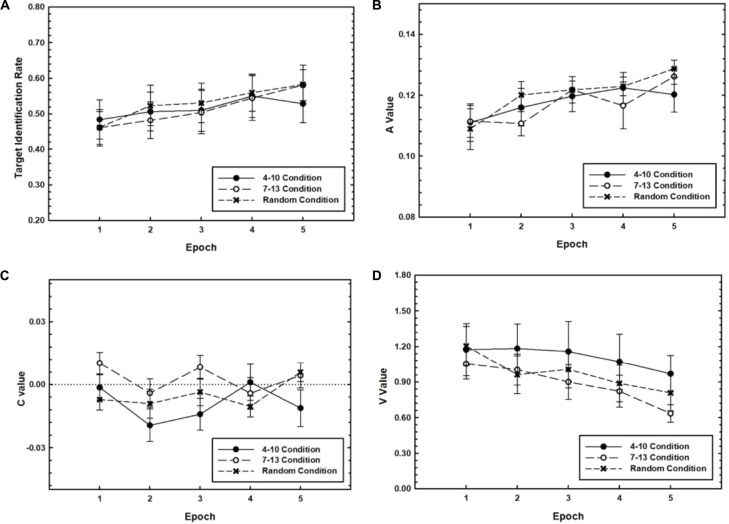
Target identification rate **(A)**, *A* values **(B)**, *C* values **(C)**, and *V* values **(D)** for RSVP performance during the training phase for the 3-Day group plotted as a function of Training Epoch. Separate plots are shown for the 4–10, 7–13, and random Target Timing conditions. Error bars represent the SEM.

We first sought to characterize the general shape of learning that took place over the extensive period of 3 days. Data from the training phase of the 3-Day condition were used to examine changes in target identification rate and *A*, *C*, and *V* values as a function of practice. We expected target identification rate and *A* values to increase, *V* values to decrease, and *C* values to converge to zero with practice. We further expected that these practice-related improvements would be especially pronounced for Target Timing conditions where the target position was fixed during Training, that is, the 4–10 and 7–13 conditions, relative to the random condition. Blocks were grouped into *Epochs* consisting of three blocks each, creating five total Epochs of training for the 3-Day condition. Mean target identification rate and *A*, *C*, and *V* values are plotted as a function of Epoch separately for each Target Timing condition in Figure 2. A 3 (Target Timing) × 5 (Epoch) repeated measures analysis of variance (*ANOVA*) was performed on each of the measures. Because of our interest in the general shape of learning and because we did not have *a priori* predictions for individual target positions, we did not differentiate between the target positions and pooled over the Target Position variable.

First, for target identification rate, the Target Timing × Epoch interaction was not significant, *p* = 0.54. However, as expected, target identification rate increased with practice, *F*(2.91,107.80) = 10.63, *p* < 0.001, η*_*p*_^2^* = *0.22*. *Post hoc* comparisons indicate that this main effect reflected significant increases from Epoch 1 to Epoch 2, *p* = 0.009, and from Epoch 3 to Epoch 4, *p* = 0.001. Target identification rate did not differ among Target Timing conditions, *p* = 0.96.

As expected, *A* values increased with practice, as evidenced by a significant main effect of Epoch, *F*(2.53,93.85) = 12.53, *p* < 0.001, η*_*p*_^2^* = 0.25. This reflected increases in *A* value during the first half of training, that is, from Epoch 1 to Epoch 2, *p* = 0.003, and Epoch 2 to Epoch 3, *p* = 0.004, according to *post hoc* comparisons. The effects subsuming Target Timing were not reliable, *p*s > 0.2.

For *C* values, there was a main effect of Epoch, *F*(4,144) = 2.57, *p* = 0.040, η*_*p*_^2^* = 0.07, such that *C* values fluctuated around 0. Specifically, *C* values decreased from Epoch 1 to Epoch 2, *p* = 0.002, and increased from Epoch 2 to Epoch 3, *p* = 0.043. The Target Timing × Epoch interaction was also significant, *F*(8,144) = 2.20, *p* = 0.031, η*_*p*_^2^* = 0.11, reflecting differences in the amplitude of this fluctuation among Target Timing conditions.

*V* values decreased with Epoch, as predicted, *F*(2.82,98.73) = 9.87, *p* < 0.001 η*_*p*_^2^* = 0.22. Specifically, *V* values decreased during the latter half of training, that is, from Epoch 3 to Epoch 4, *p* = 0.030, as shown by *post hoc* comparisons. Effects subsuming Target Timing were not reliable, *p*s > 0.5.

In sum, the training results suggest that practice led to more accurate target identification and influenced all three dimensions of attentional dynamics; efficacy (measured by the *A* value) increased early in training as illustrated by increasing *A* values, while precision increased during later training Epochs as illustrated by decreasing *V* values. Latency, however, did not appear to consistently change with practice. With the exception of the *C* value, the learning curves were monotonic, and in some cases, assumed the form of a Power function (Newell and Rosenbloom, 1981). However, these effects of practice during the training phase were not accentuated for the conditions where the target position had been fixed relative to the random condition. Therefore, these learning effects are attributed to learning of the general aspects of the RSVP task rather than the target timing which was manipulated in this study. It is also possible that advantages of fixed timing were dependent on explicit learning, which is addressed below in the section on *Explicit Learning and Adaptation*.

### Timing Specific Learning: Transfer Performance

**FIGURE 3:**
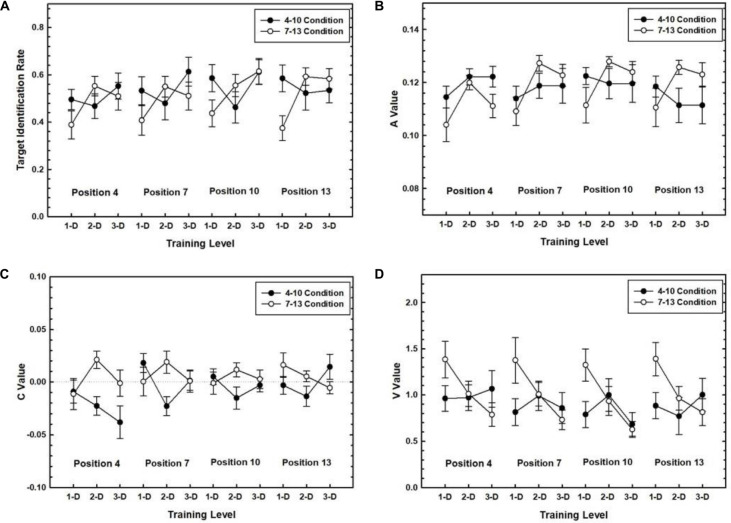
Target identification rate **(A)**, *A* values **(B)**, *C* values **(C)**, and *V* values **(D)** measuring RSVP performance in the transfer phase plotted as a function of Training Level for each of the four Target Positions. Each measure is plotted separately for the 4–10 and 7–13 Target Timing conditions. Error bars represent the SEM.

Next, to measure the degree of learning that could be attributed to the learning of the fixed target positions we focused on performance for each of the four possible target positions separately comparing conditions when a target position had been practiced during training (the practiced condition) and when it had not been practiced (the unpracticed condition). For example, we examined performance for Position 4 when Position 4 had been practiced in the 4–10 condition and compared that against when Position 4 had not been practiced in the 7–13 condition. We adopted stringent criteria for evidence for timing specific learning. Specifically, we tested whether a performance measure was greater in the practiced than in the unpracticed condition and whether this difference increased with amount of practice. Thus, in a separate 2 (Target Timing) × 3 (Training Level) ANOVA on the average target identification rate, *A* and *V* values over the three transfer blocks, we tested for interaction effects showing differences between practiced and unpracticed Target Timing conditions that increased with practice. We did not have specific predictions concerning the random condition for this analysis. Figure 3 shows each measure plotted as a function of Training Level and Target Position separately for each Target Timing condition.

The results relating to the target identification rate, *A* values, and *V* values did not reveal learning by this criterion. That is, the Target Timing × Training Level interaction was not reliable for any of these variables regardless of Target Position. The only exception was a significant Target Timing × Training Level interaction for target identification rate at Position 13, *F*(2,77) = 3.88, *p* = 0.024, η*_*p*_^2^* = 0.09. However, this interaction reflects a decrease in the gap between the 4–10 and 7–13 conditions with practice, making it difficult to infer learning occurred.

To test whether timing related learning occurred with respect to the latency of an attentional pulse, we tested whether *C* values for practiced conditions approached zero at a faster rate relative to unpracticed conditions which would be reflected in a significant Target Timing × Training Level interaction. However, *C* values for none of the Target Positions showed this pattern. The only exception was a significant Target Timing × Training Level interaction at Position 7, *F*(2,73) = 4.17, *p* = 0.019. However, this reflected both the 4–10 and 7–13 conditions similarly converging onto zero by Day 3.

Taken together, the transfer results did not show robust evidence that timing related learning occurred for any of the Target Positions.

### Explicit Learning and Adaptation

To explore the role of explicit learning in the improvement of RSVP performance and changes to attentional dynamics, we focused on the ELS computed from responses from the Explicit Learning Questionnaire. Subjects whose ELS was greater than two standard deviations from the group mean of the Target Timing and Training Level conditions were removed from the following analyses.

First, we examined the extent to which explicit learning developed regarding the temporal patterns present in the training and transfer phases.

#### ELS at Training and Transfer

**FIGURE 4:**
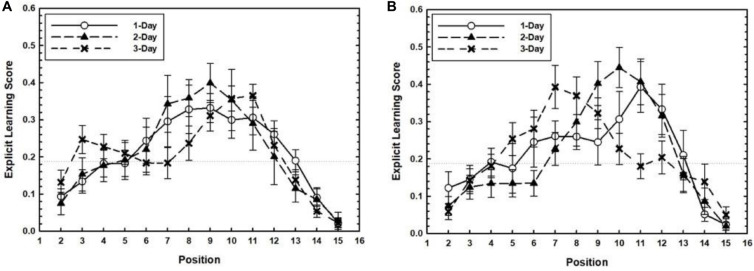
For the 4–10 **(A)** and 7–13 conditions **(B)**, the training related Explicit Learning Score (ELS) is plotted as a function of Target Position separately for each Training Level. Error bars represent the SEM.

We explored whether explicit learning about the temporal patterns during training emerged with days of practice. Training related ELS is plotted as a function of Target Position separately for each Training Level in Figure 4. Strong evidence for explicit learning of practiced target positions would be found if the ELS for practiced target positions for each Target Timing condition were greater than the non-practiced ones, especially for longer training conditions. To test this, a 2 (Target Timing: Experimental vs. Random conditions) × 3 (Training Level: 1-Day, 2-Day, and 3-Day conditions) factorial ANOVA was conducted separately for each target position. Separate analyses were conducted for each target position because explicit learning may have occurred for one of the trained positions but not for another. Participants in the 4–10 condition had larger ELS than participants in the random condition for target position 10, *F*(1,75) = 5.11, *p* = 0.027, η_*p*_^2^ = 0.06. The effects subsuming Training Level were not significant, *p*s > 0.5. Also, no effects were found for target position 4. Comparing the 7–13 condition with the random condition revealed a marginally significant effect of training level for target position 7, *F*(2,75) = 3.15, *p* = 0.048, η_*p*_^2^ = 0.07. The effects subsuming Target Timing were not significant, *p*s > 0.25. Taken together, these results did not show strong support for the emergence of explicit knowledge of practiced target positions during the training phase, although limited evidence for explicit learning of a subset of target positions could be discerned.

**FIGURE 5:**
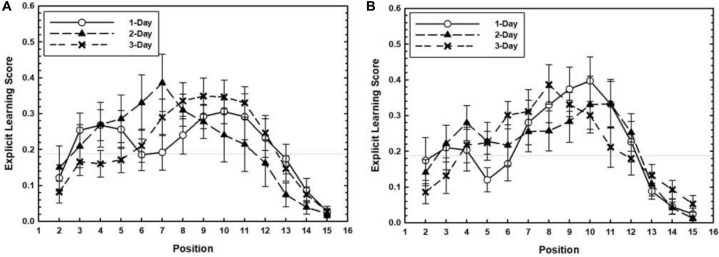
For the 4–10 **(A)** and 7–13 conditions **(B)**, the transfer related Explicit Learning Score (ELS) is plotted as a function of Target Position separately for each Training Level. Error bars represent the SEM.

In the transfer phase, both positions practiced in the training phase and newly introduced positions were presented in a repeating manner. We expected explicit knowledge for both the old and new positions to occur, due to the strengthening of explicit knowledge formed during training and the attention drawn to the positions newly introduced during transfer. In Figure 5, transfer related ELS is plotted as a function of Target Position separately for each Training Level. To test whether ELS for target positions differed among Target Timing and Training Level conditions a 3 (Training Level: 1-Day, 2-Day, and 3-Day) × 3 (Target Timing: 4–10, 7–13, and random conditions) × 4 (Target Position: Positions 4, 7, 10, and 13) mixed ANOVA was performed. Only the effect of Target Position was significant, *F*(2.41,183.12) = 12.97, *p* < 0.001, η_*p*_^2^ = 0.14 in the absence of any other main or interaction effects, *p*s > 0.3.

In sum, the group results concerning ELS in training and transfer provide only partial evidence that some explicit learning emerged in the training phase, but not the transfer phase.

#### ELS, RSVP Performance, and Measures of Attentional Dynamics

**FIGURE 6:**
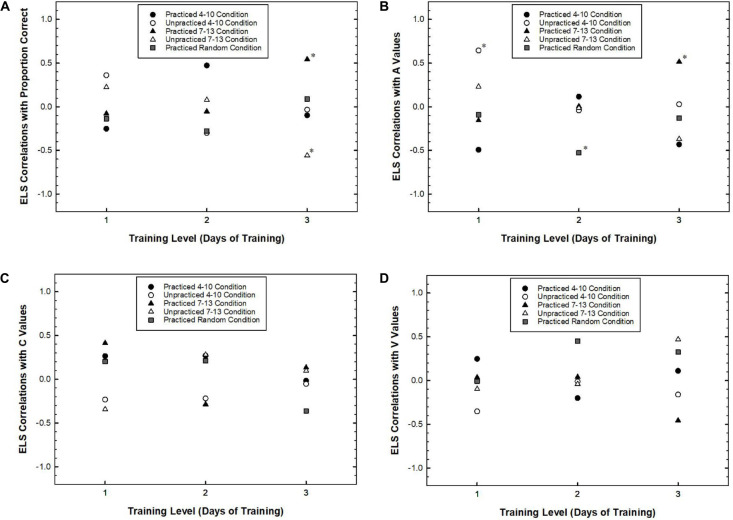
The correlation between the transfer related Explicit Learning Score (ELS) and the rate of learning for target identification rate **(A)**, *A* values **(B)**, *C* values **(C)**, and *V* values **(D)** plotted as a function of Training Level separately for practiced and unpracticed Target Positions. Asterisks represent correlations that were statistically significant (*p* < 0.05) or marginally significant (*p* < 0.08).

The results above did not reveal strong evidence that timing specific learning occurred when comparing differences between group ELS. However, some learning did occur as evidenced by changes in attentional dynamics over the course of training. One question was whether individual participants’ changes were related to their explicit knowledge for the practiced target positions. To address this question, for each participant, we conducted linear regressions on the performance measures over the course of all blocks of training. The slope of the regression function, *b*, was used as a measure of improvement. We then examined how this measure related to a measure of explicit learning. Specifically, we focused on the degree of explicit learning achieved by the end of the experiment as measured by transfer related ELS for practiced and unpracticed positions. Practiced positions are target positions that the participant experienced during training blocks and unpracticed positions are target positions that were introduced in the transfer blocks. To the extent that explicit learning of target timing had a beneficial effect on attentional adaptation, we expected that *b*, the improvement rate for each performance measure, would be positively correlated with the transfer related ELS for practiced positions. Such a relationship should not be found with respect to the transfer related ELS for unpracticed positions. Given that the transfer related ELS for practiced and unpracticed positions were partially complementary, that is, both contributing to the total of 1 for each participant, we predicted that *b* and ELS for unpracticed positions would be negatively correlated. Either pattern would support the idea that explicit learning had a beneficial effect on attentional adaptation. Figure 6 plots these correlations as a function of Training Level. Each point on the graphs represents the correlation between *b* and ELS for a Target Timing condition. That is, there are five correlations for each level of training: A correlation for practiced and unpracticed positions for both the 4–10 condition and the 7–13 condition, and a practiced correlation for the random condition. Because positions 3–14 were all practiced during training for the random condition, only one correlation between *b* for positions 4, 7, 10, and 13 and ELS for those positions is reported. Data were excluded from analyses if they matched the criteria described at the beginning of the Section “Results.”

The correlational results are largely supportive of these predictions. With respect to target identification rates for the 7–13 condition, the correlation between *b* and the ELS for the practiced positions showed an increasing trend that peaked at the 3-Day condition, *r*(11) = 0.54, *p* = 0.055. The same correlation decreased for the unpracticed positions with Training Level reaching a significantly negative correlation in the 3-Day condition, *r*(11) = −0.56, *p* = 0.047. Correlations between *b* and ELS were not significant for the 4–10 condition, *p*s > 0.10. The correlations between *b* and practiced positions for the random condition were also not significant, *p*s > 0.30.

Similarly, for *A* values, the correlation between *b* and the ELS for practiced positions in the 7–13 condition showed an increasing trend reaching a peak in the 3-Day condition, *r*(11) = 0.52, *p* = 0.072. However, the correlations with unpracticed positions for the 7–13 condition did not reach significance, *p*s > 0.20. The correlations between *b* and ELS for the 4–10 condition approached significance only for the 1-Day condition such that the correlation with unpracticed positions was positive, *r*(11) = 0.64, *p* = 0.017. Additionally, a negative correlation with practiced positions for the random condition approached significance for the 2-Day condition, *r*(12) = −0.52, *p* = 0.052.

Finally, with respect to *C* values and *V* values, none of the correlations approached statistical significance.

Taken together, these analyses support the idea that explicit learning had a facilitative effect on performance in the RSVP task as measured by proportion correct and *A* values. Interpretations of these results should be made with caution because many correlation analyses were conducted on less than 15 participants per condition. However, because correlations pertaining to the random condition were either not significant or in the opposite direction, we believe that the results concerning the conditions with fixed predictable target positions are important. In particular, the pattern of results in the 7–13 condition where correlations were near zero for participants who practiced for 1 or 2 days, but stronger for participants who practiced for 3 days suggests that explicit learning is related to attentional adjustments.

## Discussion

The enhanced deployment of selective visual attention can be inferred from the enhancement of target report in an RSVP paradigm that reflects improved perceptual and working memory processes. With practice, target identification rates for a second target in an attentional blink paradigm increase signifying a change in the way in which visual attention is distributed over the course of an RSVP trial (Choi et al., 2012; Shin et al., 2015; Willems et al., 2015). The current research extends this improvement in target identification over the course of up to three experimental sessions and over 1,500 RSVP trials in a single target RSVP task. The main goal of this study was to characterize how the temporal dynamics of an attentional pulse changed as a function of practice. We also report exploratory results concerning the relationship between explicit temporal learning, on the one hand, and RSVP performance and attentional dynamics, on the other.

The results from the training phase indicated that target identification rate steadily improved over multiple days of practice. Practice-related changes were also found with respect to attentional dynamics, where attentional efficacy and precision improved with practice. Interestingly, the time courses of improvement between efficacy and precision were dissociated, with most of the improvement in efficacy occurring in the first 2 days (500 trials) of practice and variability in the last 2 days. This difference supports the idea of Reeves and Sperling (1986) and Vul et al., (2008) positing separable dimensions of attentional dynamics. *C* values, our measure of the latency of an attentional pulse, did not change with practice in a consistent manner and instead generally fluctuated around zero throughout the course of training. Therefore, the results concerning the latency of an attentional pulse are not clear and may reflect “random fluctuation.” Together, these findings point to the possibility that the target positions were learned during the early blocks of training with attentional onset becoming modulated close to target onset before training affected the attentional efficacy and precision. These results may suggest that attentional modulation took place in stages in the order of latency, efficacy, and precision over the 3-day training period.

These patterns of results were found regardless of whether target timing was predictable, and thus, likely reflect learning that was general to this form of RSVP presentation. The results are congruent with the interpretation that the consistent stimulus presentation rate in the RSVP task enabled these adaptive adjustments in the temporal dynamics. This is in line with previous studies showing target identification could be enhanced when targets were presented in a periodic rhythm with non-target stimuli (Ronconi et al., 2016a,b).

Of central interest to this research was the degree of learning specific to the target serial positions when target positions were fixed over the course of the experiment (in the 4–10 and 7–13 conditions). Interestingly, training performance did not reveal differences among groups that could be attributed to practice with consistent target positions. Transfer performance appeared to be more sensitive to the way in which target timing was manipulated. Nevertheless, clear evidence for timing specific learning was lacking.

In this study, we assessed how the amount of practice among participant groups (1- to 3-Day Training Level conditions) were related to the emergence of explicit knowledge of target positions. The responses to the Questionnaire for Explicit Knowledge did not show strong evidence of group-wide explicit learning of the fixed target positions, although some partial knowledge appeared to have emerged. Apparently, individuals varied widely in the degree of explicit knowledge obtained. More importantly, the correlational results concerning the relationship between measures of explicit learning and the rate of improvement in the performance measures were consistent with a positive relationship between explicit learning and attentional adaptation. Specifically, larger improvement in attentional control in terms of the efficacy of an attentional pulse was associated with greater explicit knowledge of target positions. Because these results are correlational, we cannot conclude a direction for the relation between implicit learning and explicit knowledge. In fact, many accounts for the relationship between explicit and implicit learning could apply to the current results. Explicit learning may have had a facilitative effect on attentional control and RSVP performance, congruent with stage theories of skill acquisition in which automatic performance in a task follows a stage of cognitive access to internal processes (Fitts and Posner, 1967; Anderson, 1982). Conversely, attentional resources may have been freed up for explicit learning of temporal intervals when RSVP performance became highly skilled and automatic. A third possibility is that success in implicit learning triggered the explicit search for regularities (Haider and Frensch, 2009). Only further research directly manipulating the explicit learning of target positions would allow us to distinguish between these possibilities.

In conclusion, the current study provides evidence that temporal attention is adjusted with extended practice of an RSVP task. Specifically, we observed heightened efficacy characterizing a more focused attentional pulse in the time domain. These changes in the dynamics of an attentional pulse were accompanied by improved target identification performance in the RSVP task. This process of fine-tuning attention in the temporal domain extends the research on attentional fine-tuning, which previously has been concerned with the spatial domain (Posner et al., 1980; Eriksen and Yeh, 1985; Eriksen and James, 1986; Castiello and Umiltà, 1990).

## Data Availability Statement

The raw data supporting the conclusions of this article will be made available by the authors, without undue reservation.

## Ethics Statement

The studies involving human participants were reviewed and approved by Institutional Review Board of Korea University (KU-IRB-16-138-A-1). The patients/participants provided their written informed consent to participate in this study.

## Author Contributions

MJ was highly involved in the design, data analysis and graphing, and writing of the project. BP ran the experiment on all participants in their laboratory and assisted with the data analysis and writing. JS worked closely with MJ in the design, data analysis, and writing. YC provided feedback for the design, data analysis, and writing of the project. All authors contributed to the article and approved the submitted version.

## Conflict of Interest

The authors declare that the research was conducted in the absence of any commercial or financial relationships that could be construed as a potential conflict of interest.
